# Comparative Metabolomic Profiling of *Citrullus* spp. Fruits Provides Evidence for Metabolomic Divergence during Domestication

**DOI:** 10.3390/metabo11020078

**Published:** 2021-01-28

**Authors:** Pingli Yuan, Nan He, Muhammad Jawad Umer, Shengjie Zhao, Weinan Diao, Hongju Zhu, Junling Dou, Mohamed Omar Kaseb, Hanhui Kuang, Xuqiang Lu, Wenge Liu

**Affiliations:** 1 Zhengzhou Fruit Research Institute, Chinese Academy of Agricultural Sciences, Zhengzhou 450009, China; 82101179218@caas.cn (P.Y.); henan@caas.cn (N.H.); mjawadumer@gmail.com (M.J.U.); zhaoshengjie@caas.cn (S.Z.); 82101185028@caas.cn (W.D.); zhuhongju@caas.cn (H.Z.); doujunling@henau.edu.cn (J.D.); mohamedkaseb2011@gmail.com (M.O.K.); 2 College of Horticulture and Forestry Sciences, Huazhong Agricultural University, Wuhan 430070, China; kuangfile@mail.hzau.edu.cn

**Keywords:** watermelon, fruits, *Citrullus*, metabolomics, edible-seed watermelon, domestication

## Abstract

Watermelon (*Citrullus lanatus*) is one of the most nutritional fruits that is widely distributed in the whole world. The nutritional compositions are mainly influenced by the genotype and environment. However, the metabolomics of different domestication status and different flesh colors watermelon types is not fully understood. In this study, we reported an extensive assessment of metabolomic divergence in the fruit flesh among *Citrullus* sp. and within *Citrullus* sp. We demonstrate that metabolic profiling was significantly different between the wild and cultivated watermelons, the apigenin 6-C-glucoside, luteolin 6-C-glucoside, chrysoeriol C-hexoside, naringenin C-hexoside, C-pentosyl-chrysoeriol O-hexoside, and sucrose are the main divergent metabolites. Correlation analysis results revealed that flavonoids were present in one tight metabolite cluster. The main divergent metabolites in different flesh-colored cultivated watermelon fruits are p-coumaric acid, 2,3-dihydroflavone, catechin, N-(3-indolylacetyl)-l-alanine, 3,4-dihydroxycinnamic acid, and pelargonidin o-hexoside. A total of 431 differentially accumulated metabolites were identified from pairwise comparative analyses. *C. lanatus* edible-seed watermelon (cultivars) and *C. mucosospermus* (wild) have similar fruit metabolic profiles and phenotypic traits, indicating that edible-seed watermelon may be a relative of wild species and a relatively primitive differentiation type of cultivated watermelon. Our data provide extensive knowledge for metabolomics-based watermelon improvement of *Citrullus* fruits meet their enhanced nutritive properties or upgraded germplasm utility values.

## 1. Introduction

Plants synthesize an extremely large number of metabolites that have diverse structures and different functions [1,2,3,4]. Some metabolites are necessary for plant growth or reproduction; some metabolites are tightly related to abiotic and biotic responses [5,6,7]; other bioactive metabolites possess health promising properties [8,9,10,11]. Mining natural variations among various plant metabolites and exploring the metabolic networks is of great interest to scientists worldwide. The comprehensive metabolic profiling and metabolic genome-wide association study have been performed in rice, maize, tomato, wheat, and other crops, many novel genes and potential metabolic pathways have been identified and elucidated [12,13,14,15,16,17,18]. Plant metabolome is considered as a connection between the plant genome and phenotypic traits [19]. The metabolite features are closely associated with important phenotypic traits or food quality [20,21,22,23]. Thus, the specific metabolites could be used as biomarkers to select complex agronomic traits and to accelerate metabolomics-assisted breeding processes [24,25,26]. The non-targeted metabolomic-based classifications would be more probable to show correct phylogenetic relationships and may largely reflect the market-type classification [27].

Watermelon is a very important fruit worldwide and belongs to the Cucurbitaceae family. Watermelon has various genetic constitutions and abundant in nourishment for human consumption, for instance, water, lipids, sugars, organic acids, carotenoids, amino acids, fibers, nucleotides, vitamins, and others [28,29,30]. Some researchers have targeted the watermelon metabolites; 56 primary metabolites were identified in ungrafted and pumpkin-grafted watermelon using LC–ESI–MS/MS system [31]. An untargeted metabolomics study was performed on the watermelon seedlings [32]. Carotenoids in watermelons of different colors have always been a hot research topic [33,34,35]. H-NMR metabolomics method was used for the identification of metabolites among different colored (red and yellow) watermelon cultivars [35]. To date, no comprehensive metabolomics analyses have been done for different cultivated types of watermelon fruits.

Watermelon domestication was originated in Africa to be around 5000 years ago [36,37]. In genus Citrullus, *C. amarus* is a separate watermelon species type [28]. *C. mucosospermus* (seed consumption, CM, egusi) and *C. lanatus* (fruit flesh consumption) have a close phylogenetic relationship and shared ancestry [38]. The egusi in West Africa [39,40] is grown for the consumption of the seeds, and their flesh is often very bitter [41,42]. Compared with the wild ancestor, watermelon experienced substantial changes in cultivation area from tropical to various kinds of environments. The modern dessert watermelon and relative older landrace watermelon varieties are cultivated in the whole world. Edible-seed watermelon possesses a very important position for its economic seed and has been cultivated for hundreds of years in China [43]. Edible-seed watermelon is planted in an area of almost 320,000 hm^2^ and generating an annual revenue of 5.5 billion RMB, reported by Chen et al. in 2015 [44]. The cultivated watermelon experienced a significant change in plant morphological, fruit appearance, fruit nutritional composition, and growth environment. The different phenotypic characters and ecological adjustment ask for corresponding optimized metabolic pathways for plant growth and fruit development.

However, the watermelon fruit metabolome difference between various cultivated types remains poorly understood. In the present study, we assess the metabolomics differences in fruit flesh of 40 accessions that represent major types in *Citrullus*. We found that different groups of metabolites were selected for different types of watermelons during the speciation and domestication process or cultivar differentiation process. The metabolic divergence between wild and cultivated watermelon was mainly associated with the flavonoids that are good for plant growth but with bad taste. The edible-seed watermelon of China is a very special germplasm resource that could help further clarify or assess species variability, geographical differentiation, and breeding history. Our results can help to investigate the species diversity and geographical differentiation and can serve further ex situ conservation and breeding.

## 2. Results

### 2.1. Fruit Morphology of Different Types of Watermelon Fruit

**Figure 1:**
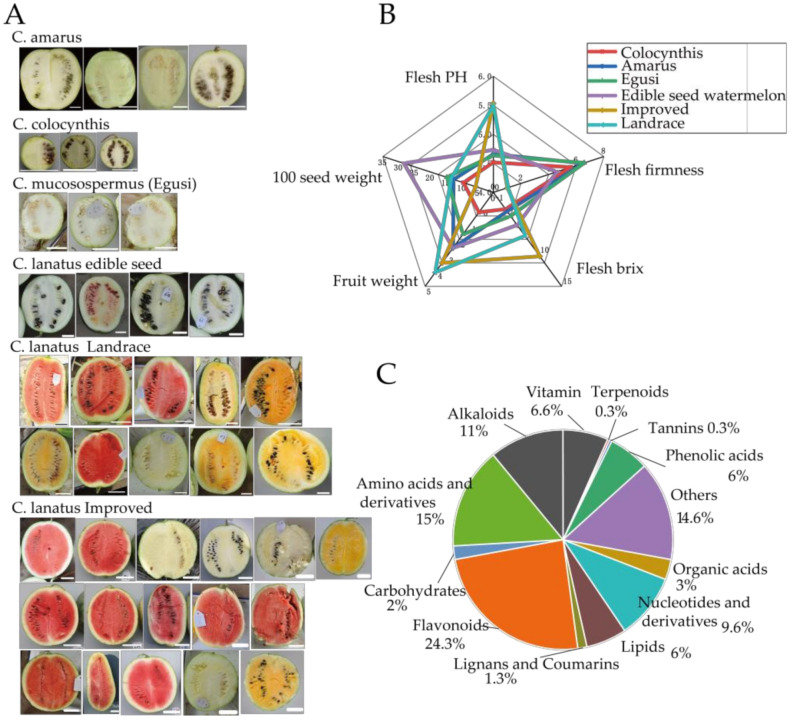
(**A**) Fruits longitudinal section image of the *Citrullus* genus in this study, organized by phylogeny [38,46,47]. (**B**) Fruit phenotypic characteristics of different types of watermelon. (**C**) Proportions of known metabolite classes identified in watermelon fruit.

Because of the high variability of color, shape, and texture among *Citrullus*, 40 representative watermelon accessions were selected, and the different visible phenotypes are shown in Figure 1A. This included 3 *C. colocynthis* (colocynthis), 4 *C. amarus* (amarus), 3 *C. mucosospermus* (egusi) [45], 4 *C. lanatus* edible-seed watermelon (edible-seed watermelon), 10 *C. lanatus* Landrace (Landrace), 16 *C. lanatus* Improved (Improved). In terms of color, 15 out of 40 accessions had a red flesh at maturity, 7 accessions had a yellow/orange flesh at maturity, while 18 accessions had white flesh at maturity. The taste of wild watermelon was bland; some wild accessions had a little bitter taste. The flesh of wild accessions was smooth, tough, and not edible. Edible-seed watermelons had a white flesh (or light red color), which is smooth, rich in polysaccharides, and feels like aloe vera juice; it is different from the sweet watermelon cultivars, which having a bright-colored flesh and crisp texture. The classification and characteristics of 40 accessions from the *Citrullus* genus used in this experiment are described in Table S1 and shown in Figure 1B.

### 2.2. Global Metabolomic Profiling in Different Types of Watermelon Fruit

#### 2.2.1. Metabolic Profiling in Different Types of Watermelon Fruit

The metabolic profiling for flesh samples of mature fruits was carried out by a widely targeted metabolome approach through liquid chromatography-mass spectrometry (LC–MS) [48], with three biological replicates. Across 40 accessions, 617 distinct metabolic traits were detected and quantified (Tables S1 and S2), and 300 metabolites were annotated. Most annotated compounds were amino acids and their derivatives, lipids, flavonoids, nucleotides and their derivatives, carbohydrates, organic acids and their derivatives, vitamins, and others (Figure 1C). All of these are essential metabolic components of the watermelon fruit. The relative contents of metabolite features were normalized (Figure S1) before the following statistical analysis. To ensure the accuracy of the measured data, the different fruit samples were mixed as quality control samples. There has a high correlation among quality control samples, and the relative standard deviation of 85.09% (525 out of 617) metabolites was below 0.3 for QC samples (Table S3), indicating the good quality of the widely targeted metabolome data.

The relative content of metabolites accumulation varied greatly among the watermelon accessions, so their genetic architecture was able to analyze effectively. Across the 40 accessions and 617 metabolites features, the average coefficient of variation (CV) was 87.11% (Figure S2A). Flavonoids showed the highest variation (123.71%). For broad-sense heritability (*H^2^*) of these metabolic features, 385 metabolites (62.40%) displayed a broad-sense heritability of greater than 0.5 (Figure S2B). Thus, it can be predicted that metabolic composition and content was primarily influenced by heritable factors.

#### 2.2.2. Clustering and Principal Component Analysis of Metabolomic Data

**Figure 2:**
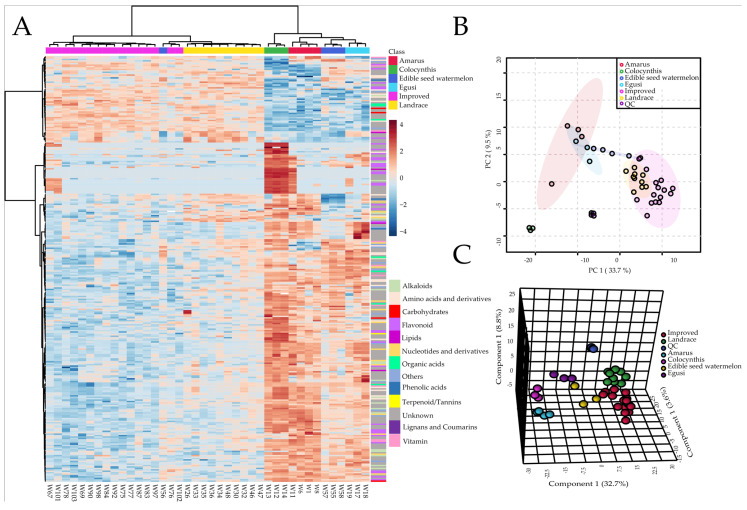
(**A**) A heatmap provides an intuitive visualization of the dataset. Forty accessions of watermelon were grouped according to 300 metabolites display the most contrasting accumulation patterns selected by ANOVA. The color of the cell corresponds to the relative concentration value according to the color scale; each column and row represents a variety and a metabolite, respectively. (**B**) Score plots of principal component analysis. (**C**) Score plots of partial least squares discriminant analysis. QC: mixed samples of different fruits flesh of various varieties.

Hierarchical cluster analysis was performed to analyze the metabolites accumulation pattern of watermelon fruit flesh in 40 core germplasm resources; these genotypes were sorted into two distinguished clusters (Figure 2A), one representing the (relatively) primitive types (wild), another cluster (cultivated) contains the landraces and improved watermelon accessions except W56 (one edible-seed variety with light red flesh). To examine the natural variations of the metabolic traits among different types of watermelon, the principal component analysis (PCA) and partial least squares discriminant analysis (PLS-DA) analysis was performed and successfully separated all the varieties into 6 clear groups (Figure 2B,C), thus clarifying the relationships among watermelon accessions. The top 10 metabolites that had the largest contribution values for PC1 are flavonoids (Table S4), of them, apigenin 6-C-glucoside, luteolin 6-C-glucoside, Chrysoeriol C-hexoside, Naringenin C-hexoside, and C-pentosyl-chrysoeriol O-hexoside, are very important metabolites, and their contents are high in the wild (*C. colocynthis, C. mucosospermus, and C. amarus*) fruits that possess bitter or bland, hard, and inedible flesh. These metabolites could probably be used as metabolite biomarkers for discrimination of the wild fruits. The top 15 important metabolites screened by PLS-DA are presented in Table S4; most of them are flavonoids, C-pentosyl-chrysoeriol O-hexoside, apigenin 6-C-glucoside, Luteolin 6-C-glucoside, naringenin C-hexoside, luteolin O-malonylhexoside, C-hexosyl-apigenin O-hexoside, delta-tridecalactone, 9-amino-1,2,3,4-tetrahydroacridine, chrysoeriol C-hexoside, gamma-dodecalactone, syringetin-3-O-glucoside, and tryptamine. The divergent metabolites between wild and cultivars are flavonoids, carbohydrates, phenolic acids, amino acids and derivatives, nucleotides and derivatives. In summary, the metabolome data generated in the current study suggests that the contents of some major metabolites in wild watermelon are quite different from that of cultivated types. Thus, it can be predicted that these metabolic features can be used to distinguish watermelon types.

In order to find certain metabolic features that are displaying particular patterns in the course of evolution and domestication, we assumed and designed a pattern of *C. colocynthis*–*C. amarus*–*C. mucosospermus*–*C. lanatus* edible-seed watermelon–*C. lanatus* Landrace–*C. lanatus* Improved [38,46,47,49], and the top 50 compounds whose levels changed based on the domestication process with high correlations were mined (Figure S3). We found out that amino acids and derivatives, flavonoids, nucleotides and derivatives, organic acid derivatives, alkaloids, and vitamins were selected during the domestication process. The α-aminoadipate, nicotianamine, O-phosphocholine, and beta-nicotinamide adenine dinucleotide gradually increased according to the hypothetical pattern. The D-pantothenic acid, pantothenic acid, tryptamine, N-hydroxy-L-tryptophan, 5-hydroxytryptophan, oxitriptan, 4-pyridoxate, theobromine, and others gradually decrease according to the pattern. We think that all the metabolites changed synergistically, leading to the emergence of modern varieties.

### 2.3. Correlation Analysis of Metabolites

**Figure 3:**
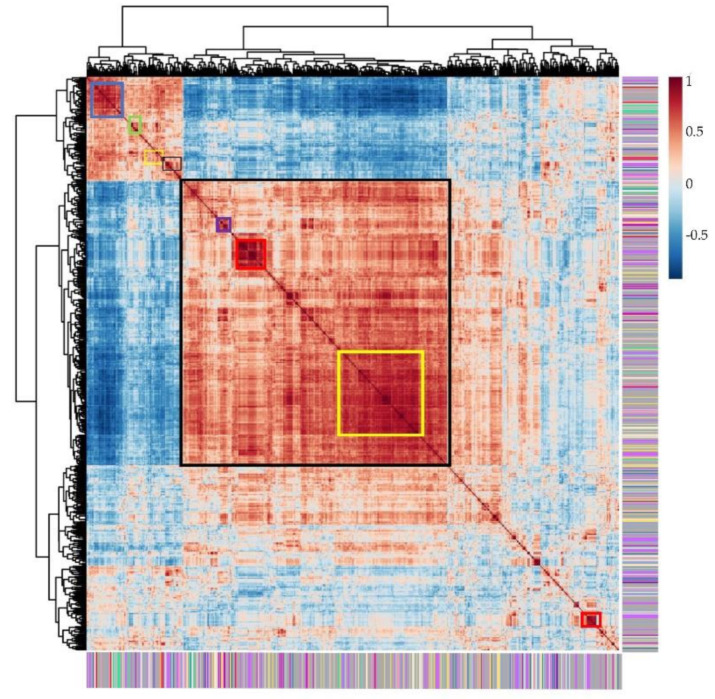
Correlation analysis of data for 617 metabolic features. The metabolites were grouped based on their accumulation patterns among 40 watermelon accessions. Each cell represents Pearson’s correlation coefficient of pairwise metabolites. The correlation coefficient value is according to the color scale. The color bins for the metabolite class are the same as in Figure 2A.

**Figure 4:**
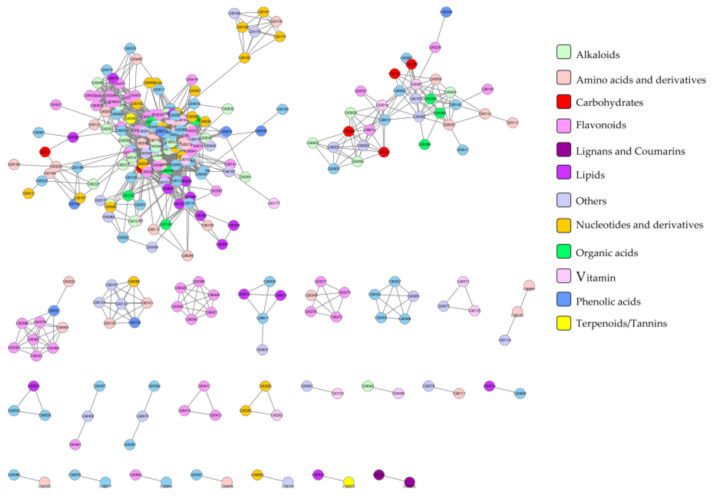
Correlation network visualization display of metabolites. The network was constructed using the paired metabolites with the coefficient index above 0.7; each node represents a metabolite, the edges connected to different nodes share similar distribution patterns among 40 watermelon accessions.

In order to reveal the relationship between various metabolites classes, correlation analysis was displayed based on the accumulation patterns of 617 detected metabolites. In Figure 3, the metabolites in rectangular blocks with different colors along the diagonal are closely related to each other, and they may have similar chemical structures, or they may be taking part in related metabolic pathways. For example, some metabolites in the blue box in the top left corner were amino acids or their derivatives, organic acids or their derivatives. Some flavonoids, alkaloids, and vitamins have a high positive correlation and are represented by the yellow box (Figure 3). The correlation metabolic network was constructed using cutoff coefficient indexes higher than 0.7 for annotated metabolites (Figure 4). Metabolites that are linked closely possibly be involved in the same or related metabolic pathways. Further inquiry on this network suggested most of the metabolites, like lipids, amino acids, nucleotide acids, organic acids, and carbohydrates, were relatively dispersed. They were found across all clusters, implying that these metabolites were very basic to support plant growth and involved in various metabolic pathways. In comparison, most of the flavonoids are grouped in the biggest cluster and three distinct subgroups, suggesting that flavonoids have unique physiological functions in the plant. Based on the correlation analysis, we can try to speculate the classes and annotation of currently unknown metabolites.

### 2.4. Comparative Metabolomics Analysis of Different Flesh-Colored Watermelon

**Figure 5:**
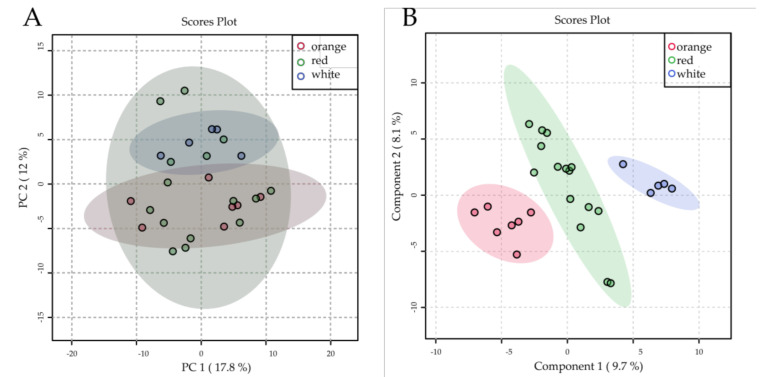
(**A**) Score plots of principal component analysis and (**B**) score plots of partial least squares discriminant analysis using data obtained from widely targeted metabolome approach for cultivated varieties.

The primitive watermelons possess white flesh, so we focus on the cultivated types (landrace and improved) for analyzing the comparative metabolomics of different flesh-colored watermelon. We also excluded three edible-seed watermelon varieties because their phenotypic and metabolomic profiles were closer to those of egusi (refer to the above result). We analyze the different colored watermelon fruits metabolome using 7 orange flesh, 15 red flesh, and 5 white flesh cultivars. The natural variations of these metabolites failed to clearly distinguish the 3 different fleshed groups by unsupervised PCA analysis model (Figure 5A), indicating relatively similar metabolomic profiling among the different colored cultivars when lacking carotenoids profiling data. For this reason, another analysis method with supervised, partial least-squares discriminant analysis (PLS-DA) was conducted to separate different flesh color watermelon varieties based on the global metabolic dataset (Figure 5B) according to the first two components (Figure S4). The very important features were filtered based on the variable importance in projection value (VIP); they are p-coumaric acid, 2,3-dihydroflavone, catechin, N-(3-indolylacetyl)-L-alanine, 3,4-dihydroxycinnamic acid, and pelargonidin O-hexoside and others (Figure S5). They are important for fruit quality and play an essential role in determining the color, appearance, flavor, and fruit taste [50]. They are also involved in many biological functions, such as antioxidant activities, enhancing immune function, anticancer, antiaging, anti-inflammatory, lowering blood pressure, and so on [51].

### 2.5. Metabolic Divergence between Different Citrullus Types

#### 2.5.1. Metabolic Divergence Based on the Domestication Process

**Figure 6:**
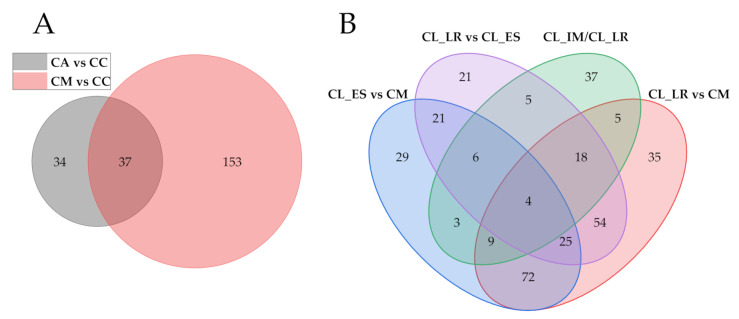
(**A**) The overlapping and unique divergent metabolites amongst the CC, CA, and CM. (**B**) The overlapping and unique divergent metabolites amongst the CL_IM, CL_LR, CL_ES, and CM. CC, CA, CM, CL_ES, CL_LR and CL_IM are *C. colocynthis*, *C. amarus*, *C. mucosospermus* (egusi), *C. lanatus* edible-seed watermelon, *C. lanatus* landrace watermelon and *C. lanatus* improved watermelon, respectively.

Different watermelon types represent a different domestication status, which is a result of human preferences or environmental influences. The divergent metabolites were screened with VIP value >1 and |fold change| ≥2 using the PLS-DA analysis module of pairwise comparative analyses (Table S5, Figure 6). The calculated results show that a total of 431 different metabolites were identified. 71 and 190 divergent metabolites were selected by comparing *C. amarus* vs. *C. colocynthis* and *C. mucosospermus* vs. *C. colocynthis*, respectively, including flavonoids, amino acid derivatives, nucleotide derivatives, phenolic acid, and alkaloids. These metabolites participate in the process of plant growth, assist plants to defend against pathogens and natural enemies, also give the fruit a bitter or astringent taste. It is worth noting that neither *C. colocynthis* nor *C. mucosospermus* fruits have a sweet taste; however, the contents of sucrose in *C. mucosospermus* fruits is 8.36 times higher than that of *C. colocynthis*. No obvious differences in sucrose contents were recorded while comparing *C. colocynthis* and *C. amarus*. 169 and 222 divergent metabolites were identified for *C. mucosospermus* vs. *C. lanatus* edible-seed watermelon and *C. mucosospermus* vs. *C. lanatus* landrace watermelon, respectively. Most of these metabolites are alkaloids, amino acids and their derivatives, vitamins, phenolic acids, flavonoids, carbohydrates and other metabolites. More differentially accumulated metabolites were observed in pairwise of *C. mucosospermus* vs. *C. lanatus* landrace watermelon as compared to pairwise of *C. mucosospermus* vs. *C. lanatus* edible-seed watermelon. There are 154 and 87 differentially accumulated metabolites were identified in pairwise comparisons of edible-seed watermelon vs. landrace watermelon and landrace watermelon vs. improved watermelon, respectively (Table S5, Figure 6).

The differential accumulation metabolic features were also screened using volcano plot with the parameter of fold change >2 and *t*-test threshold <0.1, and shown in Figure S6, to confirm the divergent metabolites using another method. There are only 6 metabolites with the FDR <0.05 in CM vs. CL_ES using *t*-tests (Table S5), together with the analysis of divergent metabolites above, the results indicating very similar fruit metabolic profiling between *C. mucosospermus* and *C. lanatus* edible-seed watermelon.

#### 2.5.2. Metabolic Divergence in Edible-Seed Watermelon

**Figure 7:**
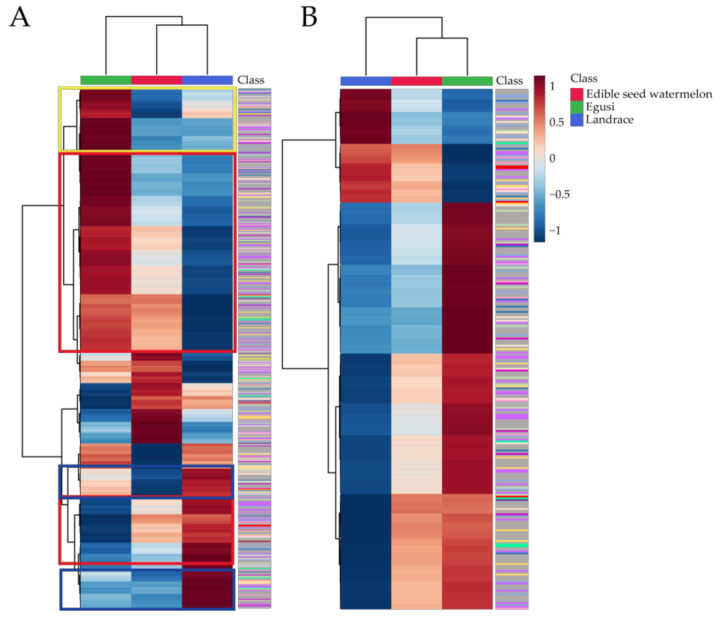
(**A**) The overall metabolomic variation trend among *C. mucosospermus* (egusi), *C. lanatus* edible-seed watermelon, and *C. lanatus* landrace watermelon (Landrace). (**B**) The heatmap of metabolites in the red box of Figure 7A. The color bins for the metabolite class are the same as in Figure 2A.

Due to the special metabolic profiling of edible-seed watermelon, we decided to further analyze the metabolomic differences among *C. mucosospermus*, *C. lanatus* edible-seed watermelon, and *C. lanatus* landrace watermelon. The overall variation trend is shown in Figure 7A. The metabolites in the red box (Figure 7A) showed a pattern of gradual increase or decrease in contents among *C. mucosospermus*–*C. lanatus* edible-seed watermelon–*C. lanatus* landrace watermelon (Figure 7B), they are vitamin, phenolic acids, organic acids and derivatives, nucleotide and derivatives, lipids, flavonoids, amino acids and derivatives, and alkaloids. These metabolites are gradually selected and changed during the process of breeding. The metabolites in the yellow and blue boxes (Figure 7A) represents the metabolites specific accumulation in egusi type or landrace type of watermelons; the metabolites are shown in the middle part of the heatmap (Figure 7A) are highest or lowest accumulated in the edible-seed watermelon. These may be related to the special living environment: egusi grew in hot and dry Africa, edible-seed watermelon in the dry and barren region (northwest of China), landrace in the different parts of the world and has been preserved by farmers for a long time. They represent specific germplasm of a certain region or locality.

## 3. Discussion

In this study, the influence of phylogeny (the result of natural and human selection) on determining fruit flesh composition was reported. The metabolic variations among a diverse watermelon population containing the 6 major types (cultivated and their wild) were systematically investigated. Whether dessert watermelons are domesticated from *cordophanus*-like populations in Northeast Africa or *mucosospermus*-like populations in west Africa [28], here, we point out edible-seed watermelon type may be an important type of watermelon. This will be useful to check over the phylogenetic relationships among species in the *Citrullus* genus taking all species and cultivated types into account [46].

### 3.1. Fruit Metabolic Difference Based on Phylogeny

Phylogenic variation be reflected by chemical composition or metabolomic characterization [27,52,53]. According to previous reports, teosinte, tropical maize and temperate maize exhibited significant divergences in distinct sets of metabolites [54]. For tomato, three different cultivation types (PIM: *S. pimpinellifolium*; CER: *S. lycopersicum var. cerasiforme*; BIG: *S. lycopersicum*) have significantly different fruit metabolic composition as illustrated in the principal component analysis for all metabolites data [16]. The metabolic profilings in cultivated lettuce were different from those in *L. serriola* [55]. In this study, the divergent metabolites among different types of watermelon were identified. The main metabolic differences between wild and cultivated watermelon were found in the flavonoids, amino acids and derivatives, nucleotides and derivatives, alkaloids, organic acids and carbohydrates. The flavonoids and alkaloids are beneficial to plant growth, but have a bitter or astringent flavor. These anti-nutritional compounds in fruit gradually decreased in the process of domestication due to the modern cultivars should contain enrich phytonutrients and are under the cautious care of the farmers [16,56,57]. These anti-nutritional metabolites changed more intensely during the initial domestication processes than in the improvement phase [55,58,59]. The recent metabolite divergences were mainly related to the fruit quality and special breeding objectives that related to the carotenoids and organic acids. The metabolic divergence in different pairwise comparisons was identified. The metabolomics- assisted classification at times reflect not only the consumption type classification but also the domestication-type classification [27]. We also explored the correlation between different metabolic groups and specific flesh colors. The associated metabolites may share the same chemical structure or contribute to the same phenotypic traits [14]. Therefore, the change of fruit metabolome in watermelon evolution and domestication can be divided into two stages, inedible to bland, tasteless fruit, bland tasteless to delicious and nutritious fruit. These results provide insights into how these different types of watermelon have evolved and differentiated at the metabolomic level, as well as valuable data resources for watermelon germplasm utilization and quality breeding. The genetic variations and metabolic variations would complement each other to better understand the complexity of this genus and to explore the watermelon domestication process.

### 3.2. Metabolic Profiling of Different Flesh-Colored Watermelon

Color is the main difference in the tested cultivated watermelon accessions. In general, the carotenoid composition and contents are related to flesh color [60,61]. However, in this study, the p-coumaric acid, 2,3-dihydroflavone, catechin, N-(3-indolylacetyl)-L-alanine, 3,4-dihydroxycinnamic acid, and pelargonidin O-hexoside and others were identified as important metabolic features in different colored watermelon using PLS-DA analysis based on the widely targeted metabolome dataset (Figure 5). The result indicated that various metabolic pathways in plants are interrelated; the different colored fruits have differences not only in carotenoid content but also in many other metabolites. These color-related metabolites are important for fruit quality and responsible for fruit flavor and taste [50]. They are also involved in many biological functions, such as antioxidant, enhancing immune function, anticancer, antiaging, anti-inflammatory, lowering blood pressure [51]. Watermelon can be a functional food and help humans against various diseases [62,63,64]. The results obtained here can be used to better understand the interaction of metabolites between different colored fruits.

### 3.3. Speculation on the Evolutionary Status of Edible-Seed Watermelon

Egusi is widely cultivated in Nigeria and other West African countries and introduced to other parts of the world from here [65,66]. Egusi watermelon plants have trailing hairy vines [67], thin wines and longer internode [47]. They are adapted to hot regions with light soil and low rainfall [68]. Compared with egusi, the edible-seed watermelon was evolved from wild watermelon ancestors. The fruit rind was relatively thick, the flesh was white and had very low sugar content [69]. Now edible-seed watermelon is cultivated as an economic crop in the northwest China region (on the Silk Road), has good storage and shipping quality [43]. The edible-seed watermelon can grow on barren land and are highly drought tolerant, with many branches, thin leaves, and vines [49]. Egusi and edible-seed watermelon are all drought-tolerant, with similar plant morphology and fruit flesh. In addition, the fruit traits index, such as fruit size, flesh Brix, flesh hardness and flesh acidity (pH), of edible-seed watermelon was closer to that of egusi than other cultivars (Figure 1B, Table S6). The genome sequence of the west African egusi watermelon is very close to the dessert watermelon [69] and shared ancestry [38]. Besides agronomic phenotypic traits and molecular genetic variation, the fruit metabolome divergence is one of the most important differentiation of watermelon species. Hence, results from the current study about fruit metabolome profiling should also be taken into consideration when the classification inference. More specifically, similar metabolomic profiling between egusi watermelon type and edible-seed watermelon type was found in this study. Hence, we think that edible-seed watermelon is closer to the egusi watermelon than any other cultivar types that we used in this study. In addition, Paris speculates that the expansion of the watermelon planting area in the world is partly because it is fruit rich in water, and the watermelon fruit can be a natural flesh water source on long voyages. (https://www.nationalgeographic.com/news/2015/08/150821-watermelon-fruit-history-agriculture/). However, the storage life of sweet dessert watermelon is very short. Hence, we can infer that this type of this watermelon with good storage and transport quality for long voyages maybe egusi with non-bitter flesh, and it was formed a different cultivation type (edible-seed watermelon) for adapting to the new environments and catering to consumers’ preferences in northwest China. Furthermore, we can think that the dessert watermelon and seed-using watermelon has probably been introduced into Asia as two independent events with different purposes. We think that the edible-seed watermelon is a very special germplasm resource, which may help us understand the domestication and breeding history of watermelon.

## 4. Materials and Methods

### 4.1. Plant Materials

In this study, 40 watermelon accessions representing different germplasm types were provided by the National Mid-term Genebank for Watermelon and Melon (Zhengzhou Fruit Research Institute, Chinese Academy of Agricultural Sciences, Zhengzhou, China). The plant materials were grown at Xinxiang in Henan Province, China (35.16° N; 113.80° E), in the spring season of 2016. After germination, seeds were hand-planted in the nutritive bowl, then transplant the one-month-old watermelon seedlings into the greenhouse with the plant and row spacing of 65 cm × 1.5 m, and a randomized block design was used. All plants were self-pollinated, and the date of pollination was marked clearly on a tag. The standard agronomic management practices were carried out for all the experiments.

### 4.2. Sample Collection and Phenotypic Investigation

Nine injury-free uniform mature watermelon fruits were harvested according to the maturity attribute of each accession. The fruits were cut longitudinally, the flesh tissues of three fruits were bulked for one biological replicate, each accession with 3 replicates. The samples were fast-frozen in liquid nitrogen before preserving in an ultra-low-temperature of −80 °C refrigerator until further analysis.

Flesh soluble solid content (Brix°%) was measured at the heart area with a hand-held refractometer (ATAGO, Tokyo, Japan). Flesh pH was determined from the heart area with a PHS-3C pH meter (Shanghai Precision and Scientific Instrument Co. Ltd, Shanghai, China). Flesh firmness was measured at the heart area by using a hardness testing machine, GY-4 (Top instrument, Zhejiang, China). The heart area was defined as the region within a radius of 1.5 cm around the cross point of longitude and longitude.

### 4.3. LC–MS

The samples were extracted as described previously [48] with some modifications. The lyophilized flesh tissue was homogenized comminuted (30 Hz, 90 s) using a tissue grinder (MM400, Retsch, Haan, Germany). 100 mg powder of each sample was weighed and extracted with 1.0 mL 70% methanol. The sample was extracted for 12 h at 4 °C. During this period, the sample was vortexed every 60 min. The extracted solution was centrifugated for 12 min at 10,000 rcf (CNWBOND Carbon-GCB SPE Cartridge, 250 mg, 3 mL; ANPEL, Shanghai, China). The resulting supernatant was filtered using a filter (SCAA-104, 0.22 μm pore size; ANPEL, China) before LC–MS detection and analysis. The UPLC-ESI-MS/MS analysis system was equipped with the Shim-pack UFLC SHIMADZU CBM30A system (www.shimadzu.com.cn/), 4500 Q TRAP (Applied Biosystems, www.appliedbiosystems.com.cn/). The analytical conditions were as follows, UPLC: column, Agilent SB-C18 (1.8 µm, 2.1 mm × 100 mm); The mobile phase consisted of solvent A, pure water with 0.1% formic acid, and solvent B, acetonitrile. Sample measurements were performed with a gradient program that employed the starting conditions of 95% A, 5% B. Within 9 min, a linear gradient to 5% A, 95% B was programmed, and a composition of 5% A, 95% B was kept for 1 min. Subsequently, a composition of 95% A, 5.0% B was adjusted within 1.10 min and kept for 2.9 min. The column oven was set to 40 °C; The injection volume was 4 μL. The effluent was alternatively connected to an ESI-triple quadrupole-linear ion trap (QTRAP)-M. The qualitative and quantitative methods are referred to in the previous report [48]. The five parameters, collision energy (CE), declustering potential (DP), precursor–product ion (Q1–Q3) and retention time (RT), were considered in compound annotation, and the database MWDB from Wuhan Metware Biotechnology Co., Ltd. was used.

### 4.4. Statistical Analysis

The broad-sense heritability (*H*^2^) of each metabolic trait was calculated through one-way ANOVA; we used three biological replicates to measure the environmental effects setting the varieties as a random effect according to the equation: *H*^2^ = *Vg*/(*Vg* + *Ve*) [12]. The raw data were normalized by mean-centered and divided by the standard deviation of each variable (UV scaling: unit variance scaling). The PCA and OPLSDA, clustering, the heat map visualization and correlation coefficients analysis were analyzed using MetaboAnalyst: https://www.metaboanalyst.ca/faces/home.xhtml. Euclidean distance was used for distance measurements, Ward’s method was used for the clustering algorithm. The metabolic network construction was displayed using Cytoscape 3.7.0.

## 5. Conclusions

The divergent metabolites marking watermelon domestication and differentiation were identified. The flavonoids, amino acids and derivatives, nucleotides and derivatives, alkaloids, organic acids and carbohydrates contents among different watermelon types can be used as biomarkers. The p-coumaric acid, 2,3-dihydroflavone, catechin, N-(3-indolylacetyl)-L-alanine, 3,4-dihydroxycinnamic acid, and pelargonidin O-hexoside were identified as important metabolites in different colored watermelon. A total of 431 divergent metabolites were identified from pairwise comparative analyses. We propose that edible-seed watermelon maybe a closer relative of egusi than the landrace or improved dessert watermelons. This result will help germplasm collection, leverage breeding and conservation purposes.

## Data Availability

The data presented in this study are available in article and supplementary material.

## Supplementary Materials

The following are available online at https://www.mdpi.com/2218-1989/11/2/78/s1, Figure S1: Comparison of metabolite contents distribution before and after normalization, Figure S2: (**A**) Distribution of variation coefficients and (**B**) the broad-sense heritability of metabolites, Figure S3: Correlation pattern of the top 50 compounds correlated with the *C. colocynthis*–*C. amarus*–*C. mucosospermus*–*C. lanatus* edible-seed watermelon–*C. lanatus* Landrace–*C. lanatus* Improved. The top 50 largest absolute value of correlation coefficient in known metabolites are shown in this figure, Figure S4: Pairwise scores plots between the top 5 components by PLS-DA analysis, Figure S5: Important metabolic traits identified by PLS-DA analysis. The colored boxes on the right indicate the corresponding metabolite relative contents in each group, Figure S6: Important features selected by volcano plot with fold change threshold (x) 2 and *t-*tests threshold (y) 0.1. The red circles indicate metabolites above the threshold. CC, *C. colocynthi*s; CA, *C. amarus*; CM, *C. mucosospermus* (egusi); CL_ES, *C. lanatus edible-seed watermelon*; CL_LR, *C. lanatus* landrace watermelon; CL_IM, *C. lanatus* improved watermelon, Table S1: The information of watermelon varieties used in this study, Table S2: Scheduled MRM transitions for widely targeted metabolite analysis in watermelon fruit flesh, Table S3: Information on QC sample metrics, Table S4: A list of the most differentially accumulated metabolites in different groups, Table S5: Comparison of metabolite levels in the intergroup, Table S6: Phenotypic characteristics of different types of watermelon fruits.
