# Supplementary material for: Comparative Metabolomic Profiling of Citrullus spp. Fruits Provides Evidence for Metabolomic Divergence during Domestication
Source: Metabolites. 2021 Jan 28;11(2):78. doi: 10.3390/metabo11020078 (PMC7911689; doi:10.3390/metabo11020078)
Supplement: Supplementary file 1 [file metabolites-11-00078-s001.zip › Supplementary Files/metabolites supplementary figs 20210127.pdf]

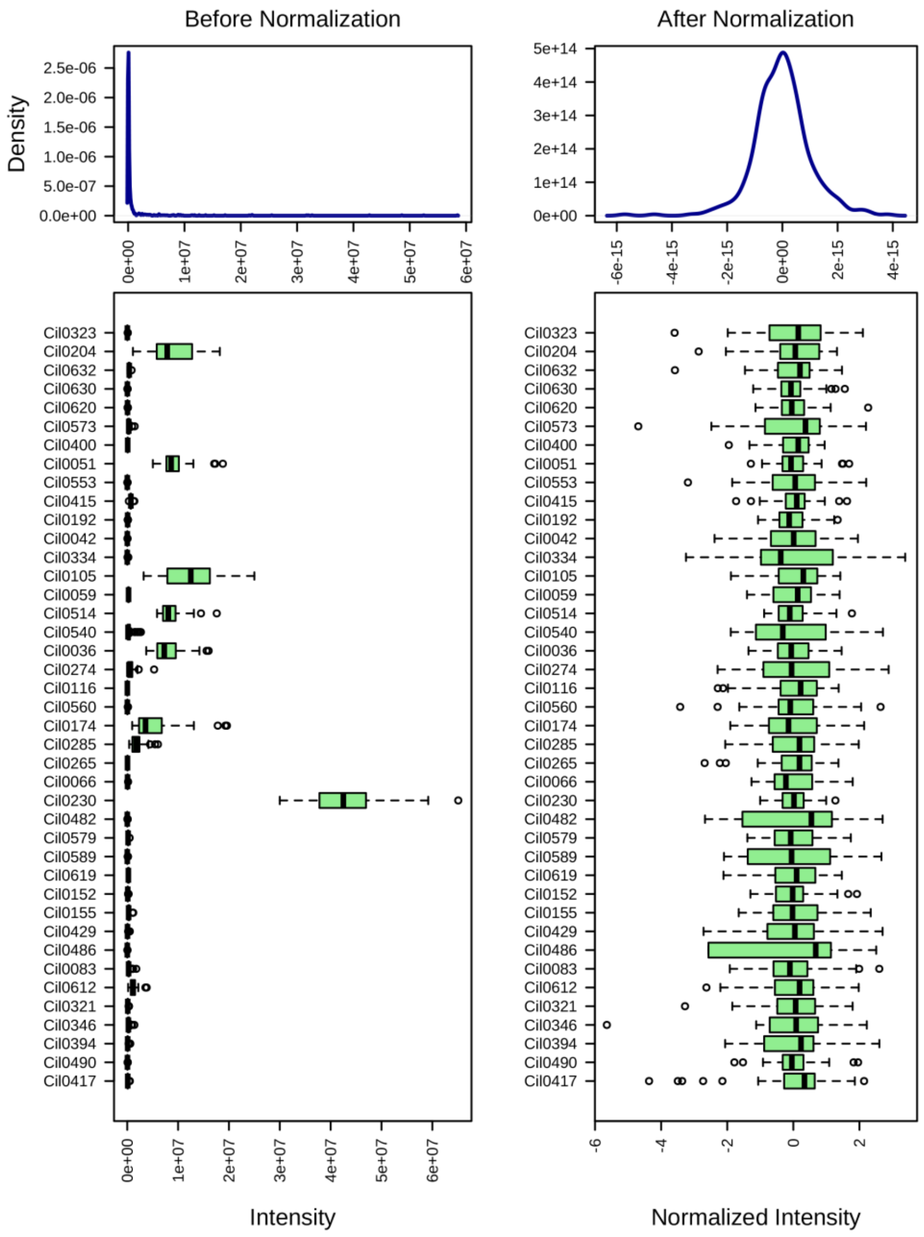

Figure S1. Comparison of metabolite contents distribution before and after normalization.

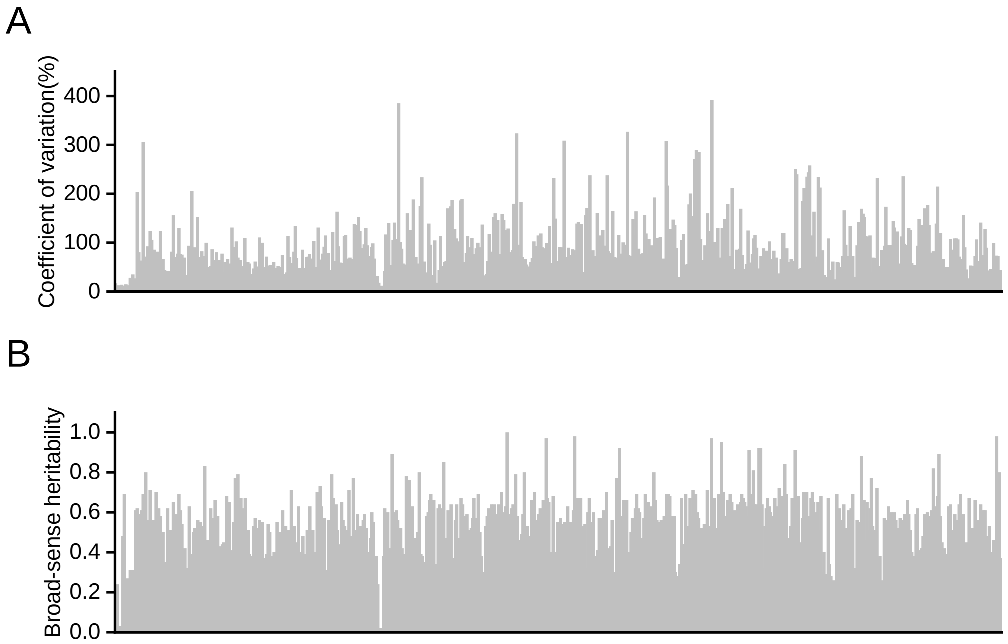

Figure S2. **(A)** Distribution of variation coefficients and **(B)** the broad-sense heritability of metabolites.

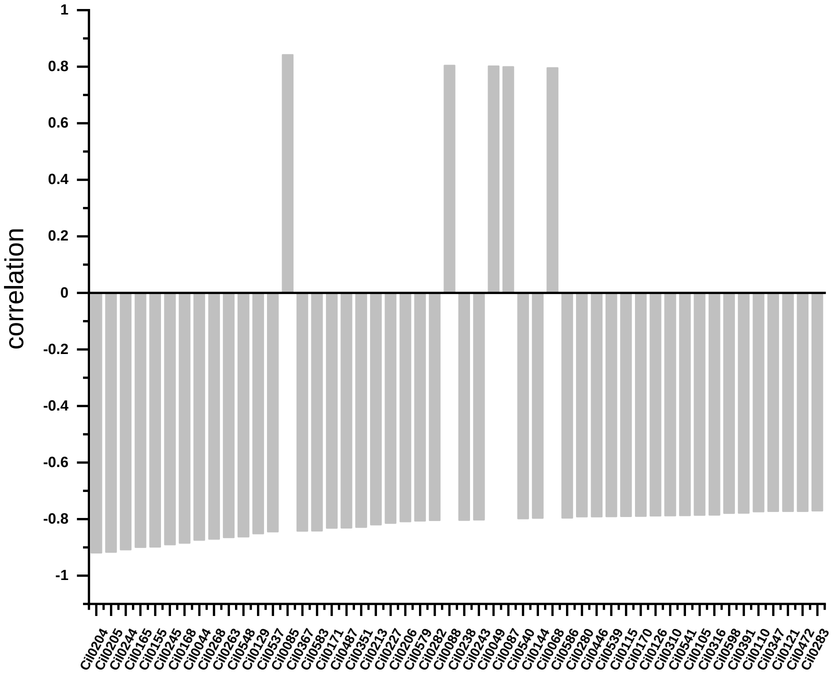

Figure S3. Correlation pattern of the top 50 compounds correlated with the *C.colocynthis* - *C.amarus* - *C. mucosospermus* - *C. lanatus* edible seed watermelon - *C. lanatus* Landrace - *C. lanatus* Improved. The top 50 largest absolute value of correlation coefficient in known metabolites are shown in this figure.

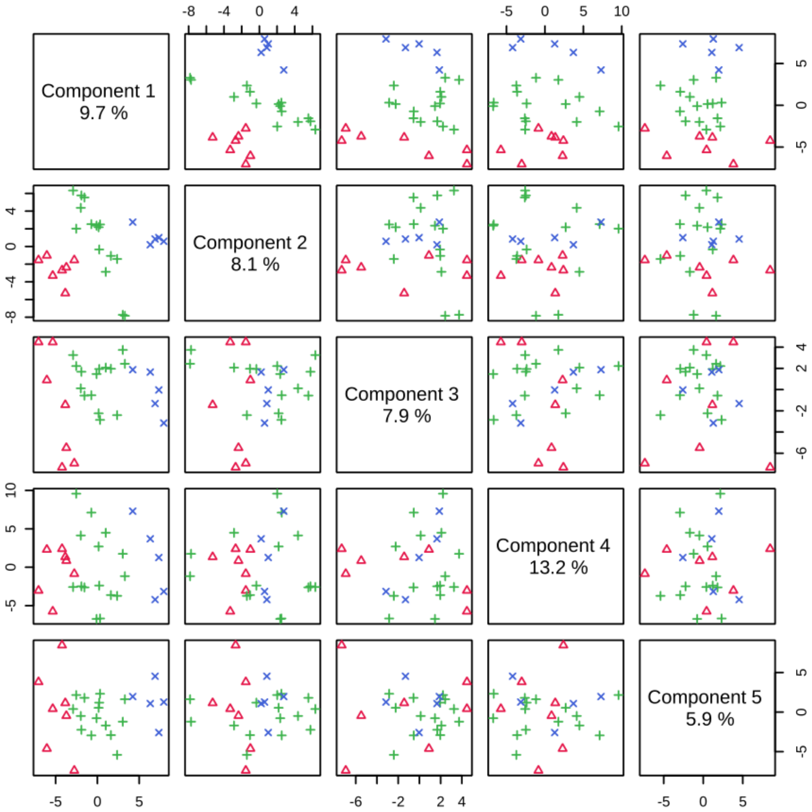

Figure S4. Pairwise scores plots between the top 5 components by PLS-DA analysis.

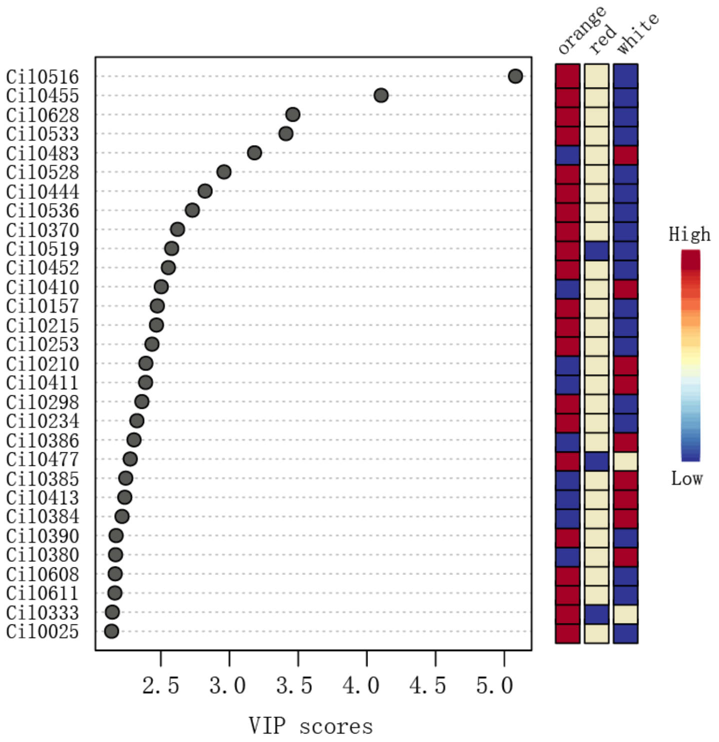

Figure S5. Important metabolic traits identified by PLS-DA analysis. The colored boxes on the right indicate the corresponding metabolite relative contents in each group.

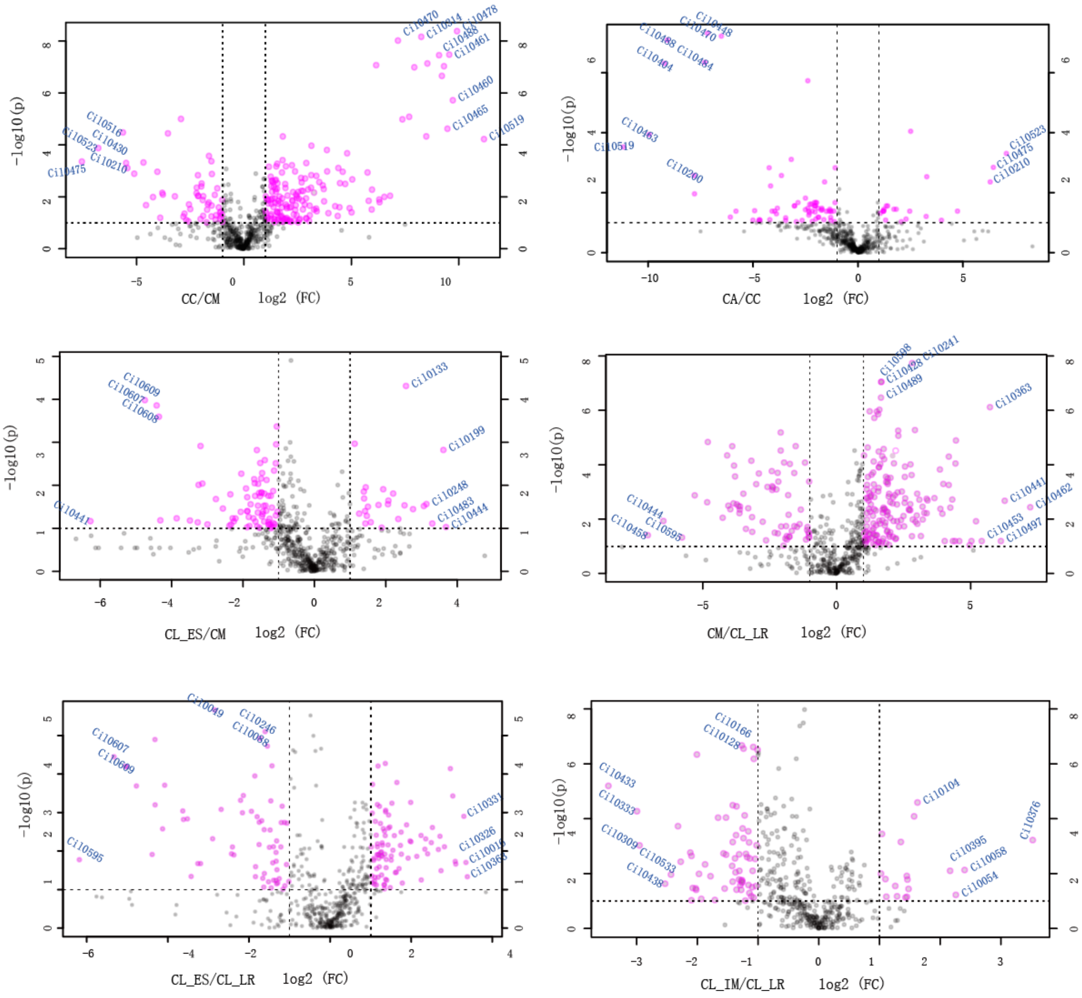

Figure S6. Important features selected by volcano plot with fold change threshold (x) 2 and t-tests threshold (y) 0.1. The red circles indicate metabolites above the threshold. CC, *C. colocynthis* ; CA, *C. amarus*; CM, *C. mucosospermus* (egusi); CL_ES, *C. lanatus edible seed watermelon*; CL_LR, *C. lanatus* landrace watermelon; CL_IM, *C. lanatus* improved watermelon.
